# Host switching is the main driver of coevolution between *Hepatozoon* parasites and their vertebrate hosts

**DOI:** 10.1186/s13071-025-06870-4

**Published:** 2025-07-23

**Authors:** Raúl Matamoros-Suárez, Víctor M. Montenegro, Federico Villalobos-Brenes, Mar Llaberia-Robledillo, Alberto Solano-Barquero, Gad Baneth, Juan Antonio Balbuena, Alicia Rojas

**Affiliations:** 1 Laboratorio de Helmintología, Facultad de Microbiología, University of Costa Rica, San José, Costa Rica; 2 Posgrado Regional en Ciencias Veterinarias Tropicales, School of Veterinary Medicine, National University of Costa Rica, Heredia, Costa Rica; 3 Laboratorio de Parasitología, School of Veterinary Medicine, National University of Costa Rica, Heredia, Costa Rica; 4 Laboratorio de Sistemática, Genética y Evolución (LabSGE), School of Biological Sciences, National University of Costa Rica, Heredia, Costa Rica; 5 Symbiosis Laboratory, Cavanilles Institute of Biodiversity and Evolutionary Biology, University of Valencia, Valencia, Spain; 6 Centro de Investigación en Enfermedades Tropicales, University of Costa Rica, San José, Costa Rica; 7 Koret School of Veterinary Medicine, The Hebrew University of Jerusalem, Rehovot, Israel

**Keywords:** Protozoa, Host, Phylogenetics, Cospeciation, Coevolution, PACo, ParaFit, EMPRess

## Abstract

**Background:**

*Hepatozoon* spp. are apicomplexan parasites with a heteroxenous life cycles, involving vertebrate intermediate hosts and invertebrate definitive hosts. These parasites infect a wide variety of wild and domestic vertebrates causing subclinical infection or mild-to-severe clinical manifestations, depending on the parasite species and vertebrate host. Interestingly, each *Hepatozoon* spp. have a specific host range, suggesting a close host–parasite coevolutionary relationship.

**Methods:**

*Hepatozoon* sequences deposited between 2013 and 2023 were mined from GenBank to test which was the most employed marker for this parasite. We reconstructed the host and parasite phylogenies using 18S rDNA and cytB sequences, respectively. Subsequent analyses were stratified according to host vertebrate orders (Carnivora, Rodentia, and Squamata), and the corresponding sequences of their *Hepatozoon* parasites. Then, Procrustean Approach to Cophylogeny (PACo) and ParaFit were employed to assess their global cophylogenetic relationships. In addition, eMPRess was used to estimate the most probable co-evolutionary events, such as host switch, duplication, sorting, or cospeciation, accounting for the shared evolutionary history of *Hepatozoon* spp. and their vertebrate hosts.

**Results:**

Global assessments of congruence between phylogenies of carnivore, rodent, and squamate hosts and those of their *Hepatozoon* parasites were significant (PACo: all *m*^2^_XY_ < 0.655, all *P* < 0.001; ParaFit: all ParaFitGlobal Statistics < 72.992, all *P* < 0.007, all Procrustes *R*^2^ > 0.25), but not for the association between *Hepatozoon* spp. and invertebrates (PACo *m*^2^_XY_ = 0.632, *P* < 0.001; ParaFitGlobal Statistic = 8.810, *P* = 0.124, *R*^2^ = 0.37). The most significant links occurred between *Hepatozoon felis* and felid hosts or *Hepatozoon canis* and canid hosts, but not between *Hepatozoon americanum* and domestic dogs or coyotes. Moreover, eMPRess showed that the coevolutionary history between *Hepatozoon* spp. and vertebrate host phylogenies was mainly explained by host switching and less frequently by cospeciation events.

**Conclusions:**

These findings highlight the ability of *Hepatozoon* spp. associated to certain vertebrate orders to infect those with a close phylogenetic relationship. This in turn helps to understand how hepatozoonosis can emerge in susceptible hosts within specific geographical areas by spillover events.

**Graphical abstract:**

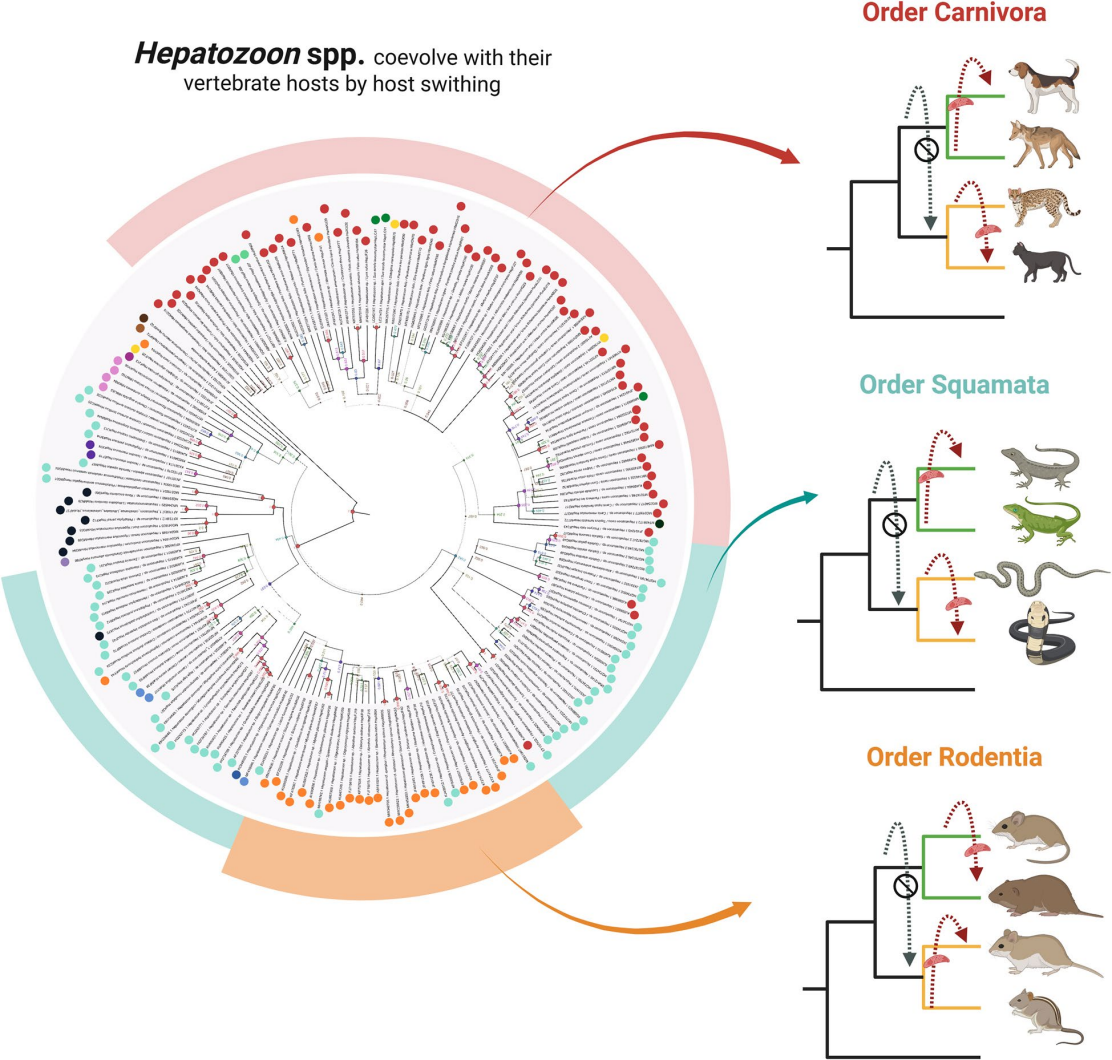

**Supplementary Information:**

The online version contains supplementary material available at 10.1186/s13071-025-06870-4.

## Background

The genus *Hepatozoon* [1] comprises more than 330 parasite species within the phylum Apicomplexa [2, 3]. These parasites infect blood cells of vertebrate hosts, both in domestic animals, such as cats and dogs, and in wild animals, including amphibians, reptiles, birds, and a variety of carnivorous mammals [4, 5]. *Hepatozoon* spp. have a heteroxenous life cycle, with asexual multiplication occurring in the vertebrate intermediate host and sexual reproduction taking place in the invertebrate definitive host [6]. Ticks and mosquitoes are the main invertebrate hosts, but mites, triatomines, tsetse flies, biting lice, sand flies, and fleas can also transmit certain *Hepatozoon* spp. to vertebrate hosts [3, 6].

*Hepatozoon* spp. can be transmitted to the vertebrate intermediate hosts via ingestion of invertebrate hosts with oocysts, consumption of prey harboring infected ectoparasites or, in mammals, by licking their coat and swallowing infected ectoparasites [5], or alternatively by vertical transmission [7]. In addition, snakes can acquire the parasite by feeding on prey infected with *Hepatozoon* meronts found in tissues, such as those found in frogs and lizards, which in turn serve as primary intermediate hosts [3, 8]. Interestingly, congenital transmission has also been reported in a brood of the banded water snake, *Nerodia fasciata confluens* upon observing gamonts in their blood [9].

*Hepatozoon* spp. have a specific host range and usually lead to subclinical or mild infections, suggesting a close host–parasite coevolutionary relationship because the parasite can adapt to host-imposed challenges [10, 11]. For instance, *Hepatozoon felis* can infect both domestic and wild cats, such as *Felis catus*, *Felis silvestris*, *Panthera pardus*, and *Panthera leo* [12, 13], and in domestic cats it mostly leads to subclinical infections with a low parasitemia and intracellular gamonts in neutrophils and monocytes [14]. In addition, *H. felis* has been suggested as a species complex since the description of *Hepatozoon ingwe* and *Hepatozoon luiperdjie* in leopards [15]. Moreover, *Hepatozoon canis* infects domestic dogs, but wild canids, such as *Vulpes vulpes*, *Cerdocyon thous*, *Lupulella mesomelas* (syn. *Canis mesomelas*), *Canis aureus*, *Canis lupus*, and *Lycaon pictus* have also been reported as hosts [16–20]. *Hepatozoon canis* infection is usually subclinical in domestic dogs, unless there is a high parasitemia or coinfection with other pathogens [21]. On the contrary, *H. americanum* induces a highly pathogenic disease with neutrophilia, muscle pain, and periosteal proliferation [22], and has been reported in domestic dogs as well as in wild coyotes [23, 24]. Therefore, the coevolutionary history between some *Hepatozoon* spp. and their hosts is uncertain because it differs between taxonomic entities, with different levels of adaptation to their hosts.

Host–parasite coevolution can be analyzed by evaluating the congruence between host and parasite phylogenies (i.e., global-fit methods) or by estimating the possible coevolutionary events that may account for the current host–parasite associations (i.e., event-based methods) [25]. In the former, the phylogenies of hosts and parasites are tangled or superposed to evaluate to which extent the phylogeny of the host mirrors that of the parasite [26]. High congruence between both phylogenies can be explained by cospeciation, in which parasites and their hosts undergo concomitant speciation due to their parallel divergence, leading to the emergence of new species for both [25, 27]. In addition to cospeciation, other coevolutionary events, such as host switching, duplication or divergence of parasites in the same host, and loss of a parasite when hosts diverge, may also occur and their frequency can be estimated using event-based methods [28].

Both approaches rely on accurate phylogenetic reconstructions of the taxa under study. Thus, the availability of high-quality molecular data is critical. In the past, when DNA sequencing and phylogenetic analyses were not available, the description of *Hepatozoon* spp. was often based on morphological and host-related features [3]. However, with the advent of molecular techniques, several phylogenetic associations have been elucidated. Consequently, some species have been transferred to other hemogregarine genera, such as *Haemolivia* or *Haemogregarina*, and conversely, some hemogregarine species have been reclassified and transferred from other genera into *Hepatozoon* [3, 29]. Furthermore, several studies indicate that the *Hepatozoon* genus is paraphyletic and consequently splitting into multiple genera has been proposed to ensure a more accurate representation of their evolutionary relationships [29, 30].

Unlike other vector-borne parasites, no cophylogenetic studies have been conducted to date to study the evolutionary history of *Hepatozoon* spp. and their various vertebrate and invertebrate hosts. In this study, we used both global-fit and event-based methods on estimated phylogenies to assess the cophylogenetic association between *Hepatozoon* spp. and their hosts, and to identify the possible coevolutionary events shaping their shared evolutionary history. Through this analysis, we expect to obtain a deeper understanding of the ecological and evolutionary determinants of these host–pathogen interactions.

## Methods

### Search strategy and selection criteria

**Fig. 1:**
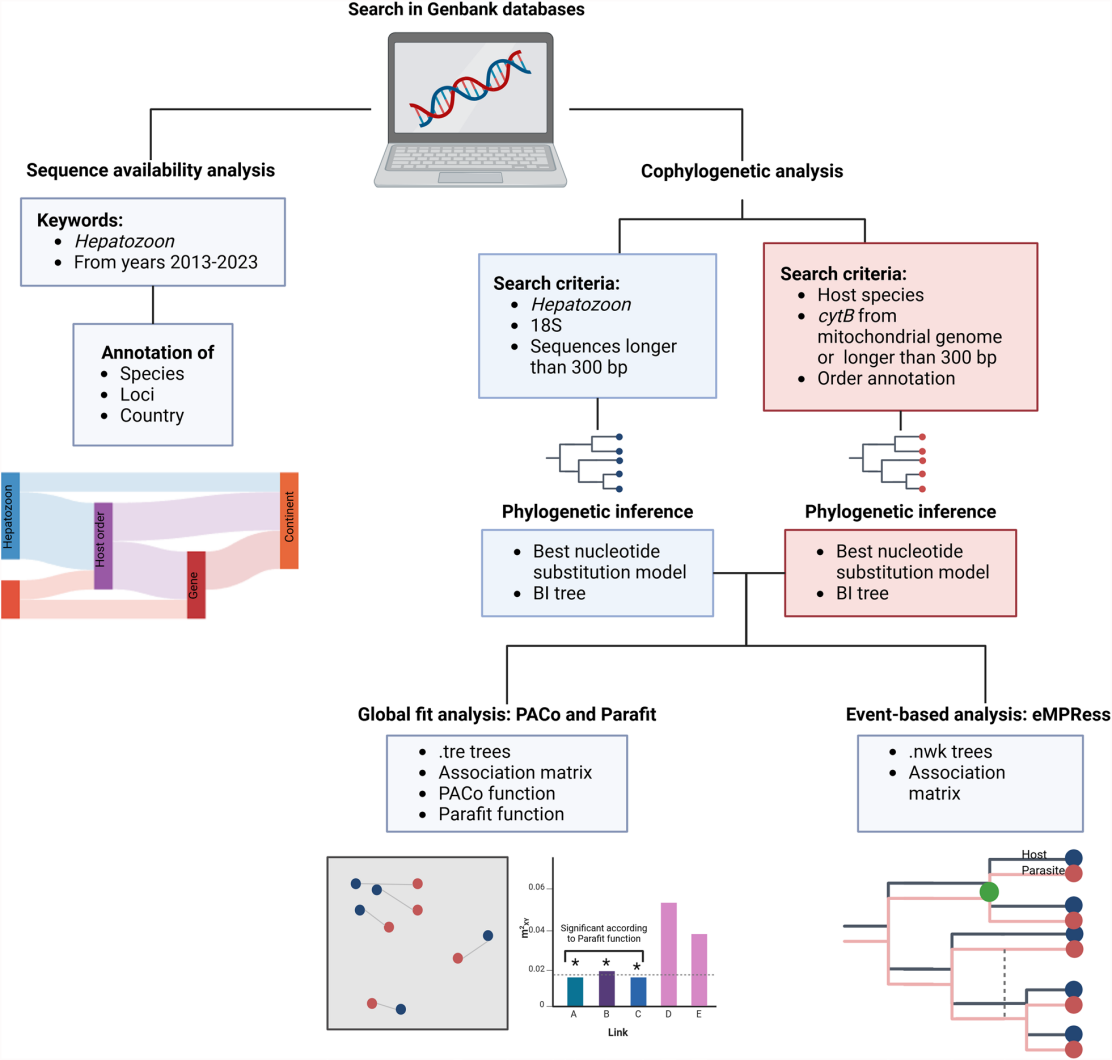
Pipeline of the analysis ran in the present study

The complete pipeline of the analysis is represented in Fig. 1. A comprehensive search of *Hepatozoon* spp. sequences deposited in GenBank between 2013 and 2023 was carried out to determine which markers have been most frequently used for this genus. In addition, the species, the host, and country in which such sequences were amplified were registered in a database and visualized using a Sankey plot [29]. The small ribosomal subunit (18S) gene of *Hepatozoon* spp. was selected for building the parasite phylogenies since it has holds enough interspecific variation and has been widely used for genotyping [30]. Likewise, the cytochrome B (*cytB*) gene was selected for estimating the hosts phylogenies owing to extensive host representation and interspecific variation. The use of two different markers for phylogenetic comparison was necessary, as they were the only markers with sufficient representation in GenBank and adequate resolution to construct well-supported phylogenies for both the host and the symbiont. Accordingly, a second database was built with 18S rDNA sequences of *Hepatozoon* spp. (*n* = 186), and *cytB* of vertebrate (*n* = 153) and invertebrate (*n* = 18) hosts in which the presence of parasite DNA was detected. Selected sequences were longer than 300 bp for both genes, aligned as a block with other sequences, were obtained from different animal hosts and geographical regions, and were obtained from GenBank in FASTA format. Cytochrome B sequences belonging to vertebrate hosts were grouped according to the orders Carnivora, Rodentia, and Squamata.

### Phylogenetic analyses

MEGA11 [31] and BEAST v2.6.7 [32] were employed to align and reconstruct the phylogenies of parasites and their hosts. Sequences were aligned with the MUSCLE algorithm [33] and unaligned ends were removed. Gaps were treated as missing data or as a fifth base. *Adelina bambarooniae* (AF494059.1) was selected as an outgroup for the parasite phylogenetic tree, *Tapirus bairdii* (JF718880.1) as an outgroup for the Carnivora host phylogenetic tree, *Caprolagus hispidus* (AY292719.1) for Rodentia host phylogenetic tree, *Sphenodon punctatus* (AY426670.1) for the Squamata host phylogenetic tree, and *Hypsibius dujardini* (NC_014848.1) for the invertebrate host phylogenetic tree. The best nucleotide substitution model was selected using JModelTest 2.1.10 on the basis of the Akaike information criterion [34, 35]. Subsequently, phylogenetic trees were constructed with Bayesian inference (BI) running one Markov chain Monte Carlo for 10^6^ generations, and sampling tree topologies every 10^3^ generations, with a burning length of 10%. Chain convergence and effective sample sizes were evaluated using Tracer v1.7.2 [36] and the information from the phylogenetic trees was summarized with TreeAnnotator v1.8.4 [37], discarding the first 10% generated trees. The phylogenetic trees were visualized using FigTree v1.4.4 [38].

### Cophylogeny analyses

Two analyses were performed to estimate the congruence between parasite and hosts phylogenies using global fit methods: Procrustes Approach to Cophylogeny (PACo) [26] and ParaFit [42]. These methods generally indicate a cophylogenetic signal, meaning closely related parasites tend to be associated with closely related hosts, rather than strict phylogenetic congruence. However, in predominantly one-to-one interaction scenarios, patterns of cophylogenetic signal and phylogenetic congruence converge, such that they can be studied interchangeably [39]. In addition, we employed an event-based method, eMPRess [43], to estimate the most probable coevolutionary events accounting for the current associations between *Hepatozoon* spp. and their hosts. Each of these analyses were performed on four different relationships between *Hepatozoon* spp. and the following host categories, based on the available number of host–parasite associations: (i) invertebrate hosts, (ii) vertebrate hosts of the order Carnivora, (iii) vertebrate hosts of the order Squamata, and (iv) vertebrate hosts of the order Rodentia.

To implement PACo [26], a unique code was assigned for each taxon of *Hepatozoon* spp. and their hosts (Additional file 1). The input data consisted of two phylogenetic trees, one for hosts and one for parasites in Newick format, and a binary matrix in text format coding the host parasite associations, where “1” denotes association between a given host species (in rows) and a parasite species (in columns) and “0” indicates no association. PACo computed the patristic distances between each taxon, where the resulting distance matrices of the *Hepatozoon* spp. and their hosts were transformed into principal coordinate (PCo) matrices. Then, the *Hepatozoon*-PCo coordinates were superimposed onto the host PCo coordinates. Therefore, the global fit or phylogenetic congruence was controlled by the host phylogeny. To evaluate if the host and parasite phylogenies were independent, the global goodness of fit statistic (residual sum of squares of the Procrustes superimposition, *m*^2^_XY_) was calculated in symmetrical (sym = TRUE) and asymmetrical (sym = FALSE) mode. The significance was determined on the basis of 10^3^ replicates in which the host–parasite associations were randomized. The *m*^2^_XY_ value is inversely proportional to the topological congruence between the host–parasite phylogenies. When the asymmetrical mode is considered, the parasite’s phylogeny is significantly constrained by that of their hosts if the proportion of *m*^2^_XY_s computed in each of the 10^3^ randomizations is less than the observed *m*^2^_XY_. Conversely, a Procrustes *R*^2^ was calculated (1 − *m*^2^_XY_) for the symmetrical mode, where a value of *R*^2^ > 0.25 suggests a pattern of phylogenetic congruence [39]. In addition, the contribution of each host–parasite association to the global fit was estimated by computing the individual square residual and its 95% confidence interval using a jackknife procedure. PACo was implemented in R [40, 41] using ape [42] and vegan [43] packages. This analysis was done as a single test on the dataset explained above. An additional dataset was built by reducing the number of *H. felis* and *H. canis* from the complete database of *Hepatozoon* spp. associated to carnivore hosts to test the effect of the representation of these sequences in the obtained results.

ParaFit is based on the fourth-corner problem [44]. Here it was implemented with the same input files as in PACo, based on the patristic distance matrices generated from the phylogenetic trees [44]. These matrices were transformed to PCo ordinations, and the host and parasite PCo ordinations were crossed with the respective host–parasite associations. An additional matrix D of fourth-corner parameters was generated, which served to compute the ParaFitGlobal statistic. Then, the contribution of each link to the global statistic was evaluated with the derived formula of ParaFitLink1. The significance of global fit and individual link contribution was assessed by randomizing the host–parasite association matrix. The analysis was performed in R [40, 41] with function “parafit” of the ape package [42], running 9999 permutations with the Cailliez correction for negative eigenvalues.

In addition, we used the event-based method eMPRess to estimate the most probable number of evolutionary events needed to account for the current host–parasite associations given their host and parasite phylogenies [45]. The analysis was done in those host–parasite associations where significant congruence was obtained either by the PACo or ParaFit approaches, i.e., in Carnivora–*Hepatozoon*, Rodentia–*Hepatozoon*, Squamata–*Hepatozoon*, and invertebrate host–*Hepatozoon*. In this analysis, costs of loss were constant at 1.00 and cospeciation at 0.00, while costs of duplication and transfer were assigned according to the Costscape plot (Table 1; Additional file 2). These two values were chosen according to: (i) the histogram section where the highest number of most parsimonious resolutions (MPRs) was obtained, and (ii) the lowest duplication and transfer values in this histogram section. Phylogenetic trees in Newick format and the host–parasite association matrix in mapping format were used as input data. Then, superimposed host and parasite phylogenetic trees were graphed with possible coevolutionary solutions. Scripts used for running the analyses are available in Additional file 3 and the public repository Zenodo at 10.5281/zenodo.15486134.

**Table 1:** Statistics for the evaluation of congruence between parasite–host phylogeny, DTL model event costs, frequencies of specific evolutionary events involved, and the best nucleotide substitution model for reconstruction of parasite and host phylogeny

Order	Global goodness-of-fit statistic *m*^2^_XY_	*P*-value^1^	*R*^2^	ParaFitGlobal statistic	*P*-value^1^	Cost	Cospeciation	Duplication	Transfer (host switch)	Loss	Best nucleotide substitution model (parasite)	Best nucleotide substitution model (host)
Carnivora	0.5199175	< 0.001	0.4800825	72.99192	0.001	D: 1.06, T: 1.01, L: 1.00	15	1	48	6	HKY + I + G	GTR + I + G
Carnivora (reduced dataset)^a^	0.501	< 0.001	0.499	0.011	0.001	D: 1.07, T: 1.05, L: 1.00	11	1	36	3	HKY + I + G	GTR + I + G
Rodentia	0.5298977	< 0.001	0.4701023	9.635127	0.004	D: 0.54, T: 0.53, L: 1.00	9	3	17	0	TPM3uf + G	TIM3 + I + G
Squamata	0.6546337	< 0.001	0.3453663	55.03327	0.007	D: 0.53, T: 1.0, L: 1.00	10	1	48	0	TPM1uf + G	GTR + I + G
Invertebrate	0.6319949	< 0.001	0.3680051	8.810297	0.124	D: 2.08, T: 4.08, L: 1.00	8	1	10	11	HKY + G	GTR + I + G

D, event cost for cospeciation; T, event cost for transfer; L, event cost for loss

^a^ Seven *H. canis* and nine *H. felis* sequences were removed from the original Carnivora dataset

^1^*P*-value at a significance level of 0.05

## Results

### *Hepatozoon* sequence availability in Genbank

**Fig. 2:**
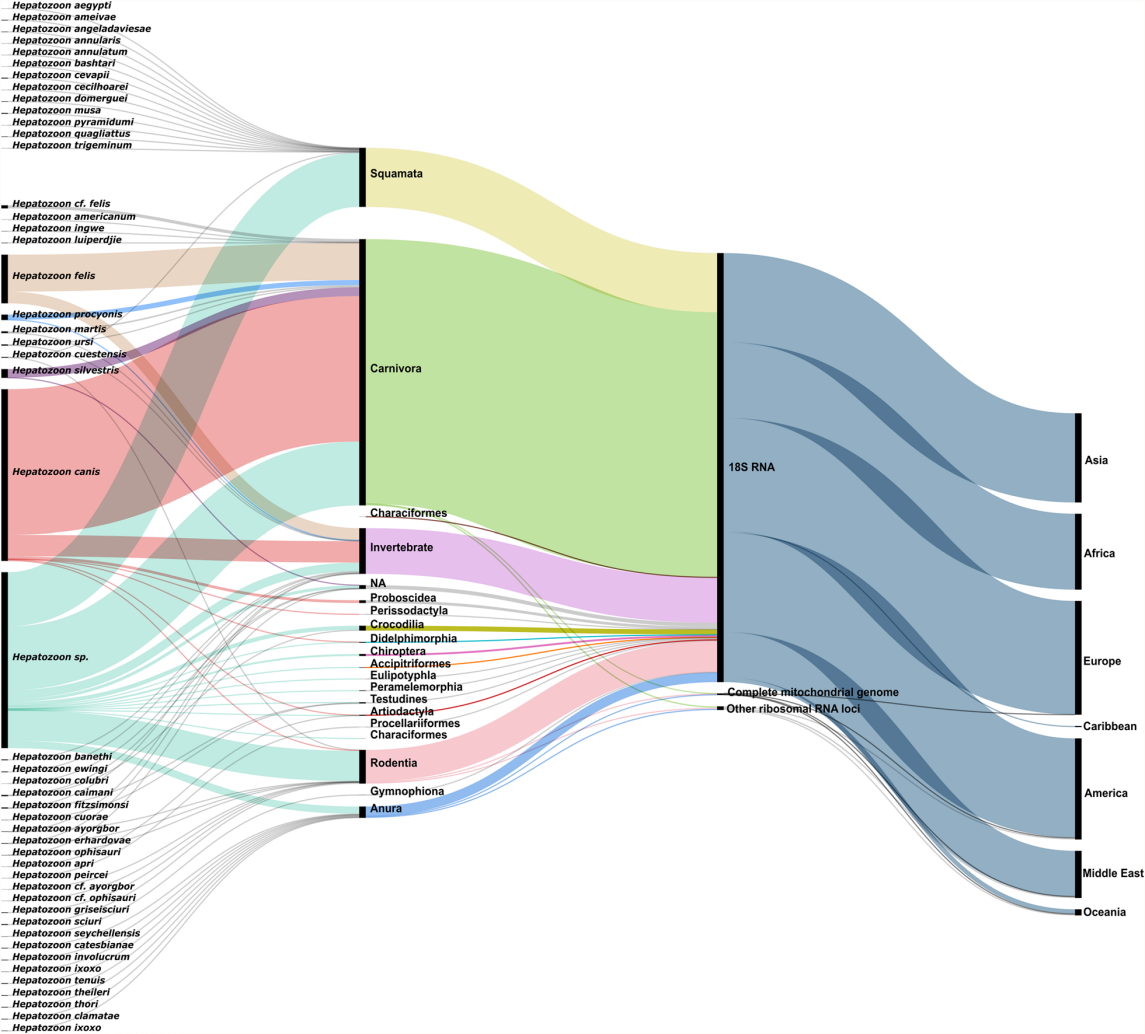
*Hepatozoon* spp. sequences deposited in GenBank from 2013 to 2023 stratified according to species, associated hosts, molecular markers, and geographical regions

A total of 3193 DNA sequences of 46 *Hepatozoon* spp. have been deposited in GenBank databases from 2013 to 2023 (Fig. 2). These sequences correspond to the markers cytochrome c oxidase subunit I (*COI*), the cytochrome c oxidase subunit III (*COIII*), the cytochrome b (*cytB*), the internal transcribed spacer 1 (ITS1), the internal transcribed spacer 2 (ITS2), the 28S ribosomal rDNA (28S rDNA), the 5.8S ribosomal rDNA (5.8S rDNA), and the 18S ribosomal rDNA (18S rDNA). 18S rDNA gene represented 99% of the sequences used in previous studies, which supports the use of this marker in the present study (Fig. 2). Most sequences correspond to *Hepatozoon* spp. from Carnivora (61.35%, *n* = 1959), followed by those from Squamata (13.62%, *n* = 435), invertebrate hosts (10.46%, *n* = 334) and Rodentia (7.77%, *n* = 248). In total, 39% of all *Hepatozoon* sequences were identified as *H. canis* (*n* = 1261). However, 40.53% of the sequences were only to genus level, i.e., as *Hepatozoon* sp. (*n* = 1294).

### Phylogeny of *Hepatozoon* spp. and their hosts

**Fig. 3:**
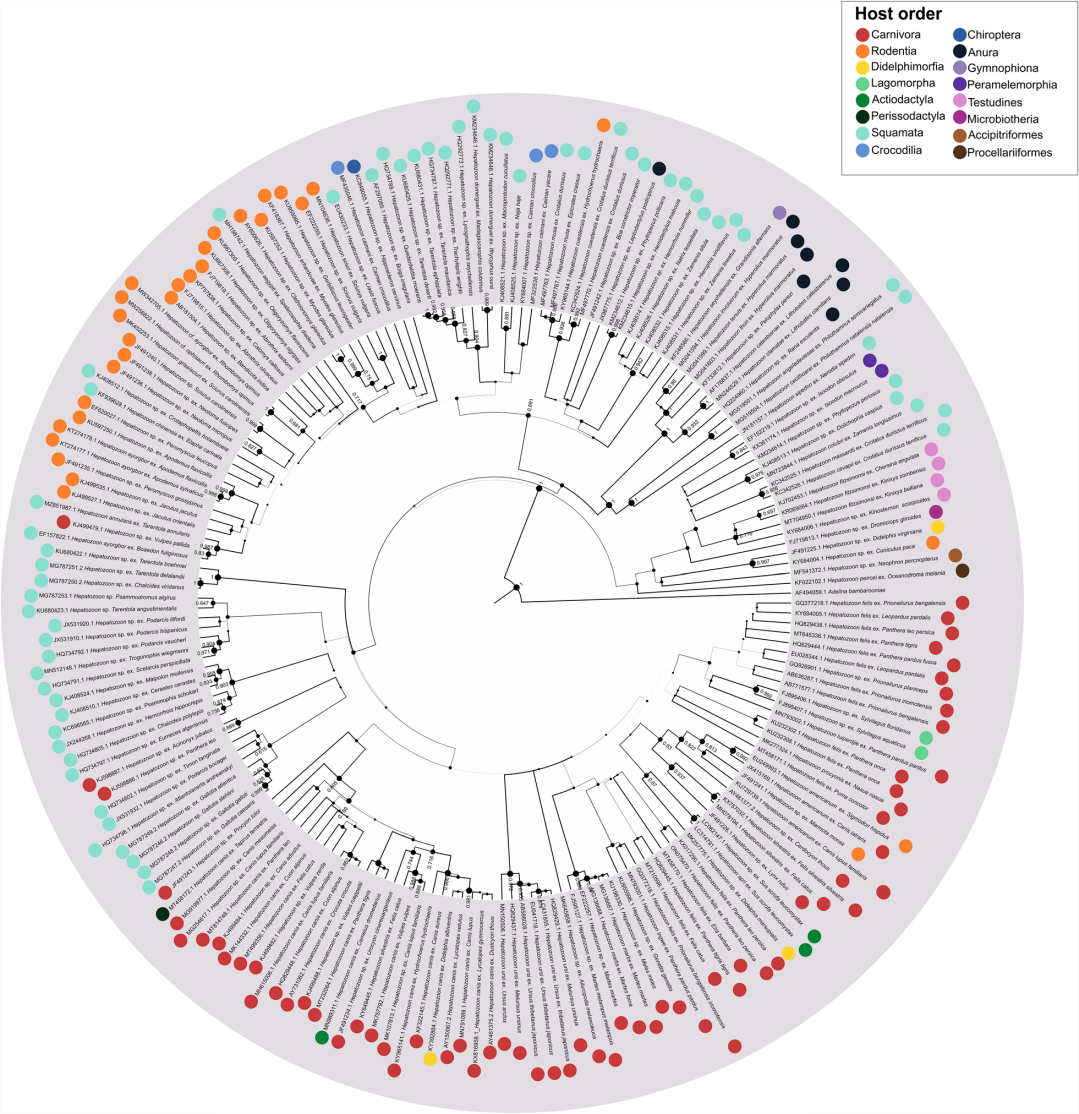
Bayesian inference phylogenetic tree of *Hepatozoon* spp. 18S rDNA sequences. Posterior probability (PP) values are denoted by node size and color. PP values lower than 0.6 are not shown in the tree. The associated host order is indicated next to each sequence

The phylogeny of *Hepatozoon* spp. was reconstructed using 175 18S rDNA sequences belonging to 39 species (Fig. 3). These sequences were retrieved from 153 vertebrate hosts as annotated in GenBank: 57 from the order Squamata, 44 from the order Carnivora, 25 from the order Rodentia, and 27 from other vertebrates (13 orders including Anura, Accipitriformes, Procellariiformes, Testudines, Gumnophiona, Chiroptera, Artiodactyla, and Lagomorpha). *Hepatozoon* sequences were also obtained from 20 invertebrate hosts: 18 from the family Ixodidae, 1 from the family Culicidae, and 1 from the family Ctenophthalmidae. Three clusters of parasite species associated with the orders Carnivora, Squamata, and Rodentia could be defined (Fig. 3). For this parasite tree, 36% of the nodes presented a bootstrap value ≥ 0.75. For the hosts, 81% of nodes from the Carnivora tree (Additional file 4), 100% of nodes from the Rodentia tree (Additional file 5), 87% of nodes from the Squamata tree (Additional file 6), and 89% of nodes from the invertebrate tree (Additional file 7) exhibited a bootstrap value ≥ 0.75.

### *Hepatozoon*–Carnivora associations

**Fig. 4:**
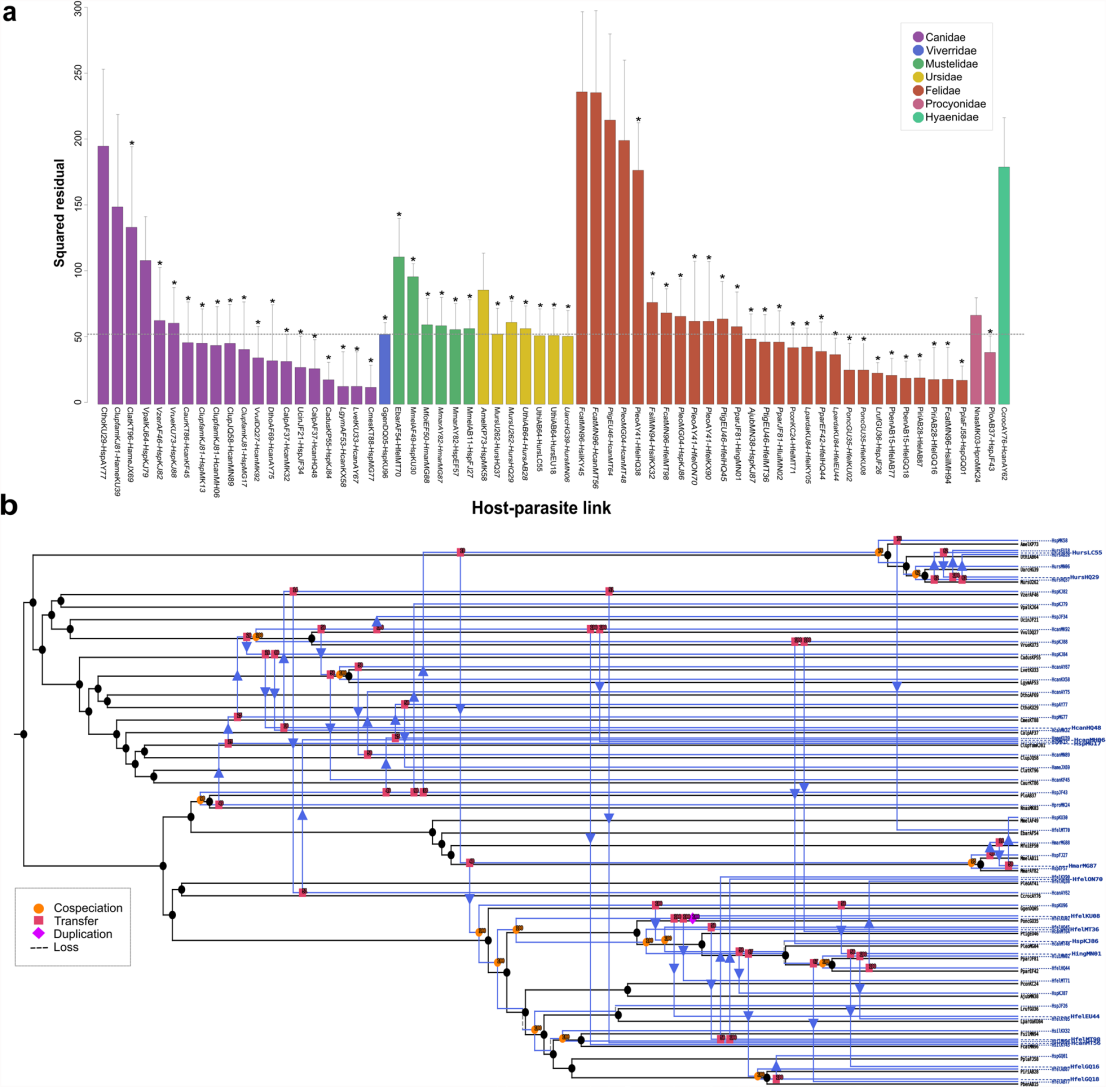
Global-fit and event-based cophylogenetic analysis between *Hepatozoon* and its carnivore hosts. **a** Contribution of each *Hepatozoon*–vertebrate host link to the global phylogenetic congruence. Each bar represents the squared residual of each association, and are color-coded according to the host family. Error bars correspond to 95% confidence intervals of the squared residuals. The median squared residual is indicated as a dotted line. Asterisks at the top of each bar represent a significant ParaFitLink1 value. **b** Coevolutionary reconstruction of the host (black lines) and parasite (blue lines) phylogenies with the lowest global cost according to eMPRess

PACo analysis of *Hepatozoon* spp. associated to hosts of the order Carnivora revealed a significant cophylogenetic relationship (*m*^2^_XY_ = 0.520, *P* < 0.001, *R*^2^ = 0.48) (Fig. 4a). Most sequences derived from carnivore hosts were uncharacterized (29.2%, *n* = 19), followed by *H. felis* (27.7%, *n* = 18), *H. canis* (20%, *n* = 13), and *H. ursi* (7.7%, *n* = 5). The 95% confidence of squared residuals of 17 host–parasite associations were below the median square residual value (Fig. 4a), with 10 of them corresponding to the family Felidae, 6 to Canidae, and 1 to Procyonidae. The associations between *Hepatozoon* spp. and Felidae included those between *Hepatozoon silvestris* with the domestic cat *Felis catus* and the wildcat *Felis silvestris*; and *H. felis* with the leopard cat *Prionailurus bengalensis*, the jaguar *Panthera onca*, and the tiger *Panthera tigris*. The links associated with the family Canidae included associations between uncharacterized *Hepatozoon* sp. sequences and the side-striped jackal *Lupulella adusta* (syn. *Canis adustus*) and the black-backed jackal *L. mesomelas*.

The highest squared residual corresponded to the cat *Felis catus* (FcatMN96) and *Hepatozoon silvestris* (HsilKY45), and the lowest concerned the hoary fox *Lycalopex vetulus* (LvetKU33) and *H. canis* (HcanAY67), and the Pampas fox *Lycalopex gymnocercus* (LgymAF53) and *H. canis* (HcanKX58). The PCo superposition plot of *Hepatozoon* spp. and Carnivora suggest three clusters corresponding to Felidae, Canidae, and Mustelidae (Additional file 4B).

The Parafit function mirrored the results obtained with PACo since a significant cophylogenetic relationship was obtained (ParaFitGlobal Statistic = 72.992, *P* = 0.001; Fig. 4a). Furthermore, 55 significant host–parasite associations were considered to contribute significantly to the cophylogenetic pattern. The individual contributions of these associations to the ParaFitGlobal Statistic indicated that the congruence between phylogenies was accounted for mostly by two families: Canidae with 17 significant links out of 20, and Felidae with 24 significant links out of 28 (Fig. 4a). The lowest ParaFitLink1 value was between the racoon *Procyon lotor* (PloAB37) and *Hepatozoon* sp. (HspJF43) (F1 = 0.54966, *P* = 0.032), and the highest was observed between the wolf *Canis lupus* (ClupJQ58) and *H. canis* (HcanMN89) (F1 = 4.94987, *P* = 0.001). Interestingly, none of the two global fit tests revealed significant associations between *H. americanum* with the domestic dog *Canis lupus familiaris* and the coyote *Canis latrans*.

eMPRess determined that host switching (*n* = 45) was the most frequent coevolutionary event, followed by cospeciation (*n* = 18) and duplication (*n* = 1) (Fig. 4b). For instance, host switches of *Hepatozoon* sp. (HspKJ88) were predicted from *Vulpes rueppelli* to *Panthera leo* and *P. tigris*. The resemblance between these two *H. felis* sequences was also observed in the *Hepatozoon* phylogenetic tree (Fig. 3). In addition, *H. felis* (HfelHQ44) from *Panthera pardus fusca* may have switched to *Panthera leo persica*, and a *Hepatozoon* sp. (HspJF43) of *Procyon lotor* to *Vulpes pallida*. Cospeciation was predicted between *H. canis* (HcanAY67/HcanKX58) with *Lycalopex gymnocercus* and *Lycalopex vetulus*, and between *Hepatozoon* sp. (HspJF43) and *Hepatozoon procyonis* (HproMK24) from *P. lotor* and *Nasua nasua*.

To evaluate the potential effect of *H. canis* and *H. felis* overrepresentation in the analysis, seven and nine sequences of these *Hepatozoon* spp., respectively, were removed from the dataset. The PACo, Parafit, and eMPRess values confirmed the results obtained with the complete *Hepatozoon*–Carnivora database, with a significant cophylogenetic signal obtained in PACo (*m*^2^_XY_ = 0.501, *P* < 0.001, *R*^2^ = 0.499) and Parafit (ParaFitGlobal Statistic = 0.011, *P* = 0.001). In addition, host switching was still the most frequent event, with 36 transfers followed by 11 cospeciation events.

### *Hepatozoon*–Rodentia associations

**Fig. 5:**
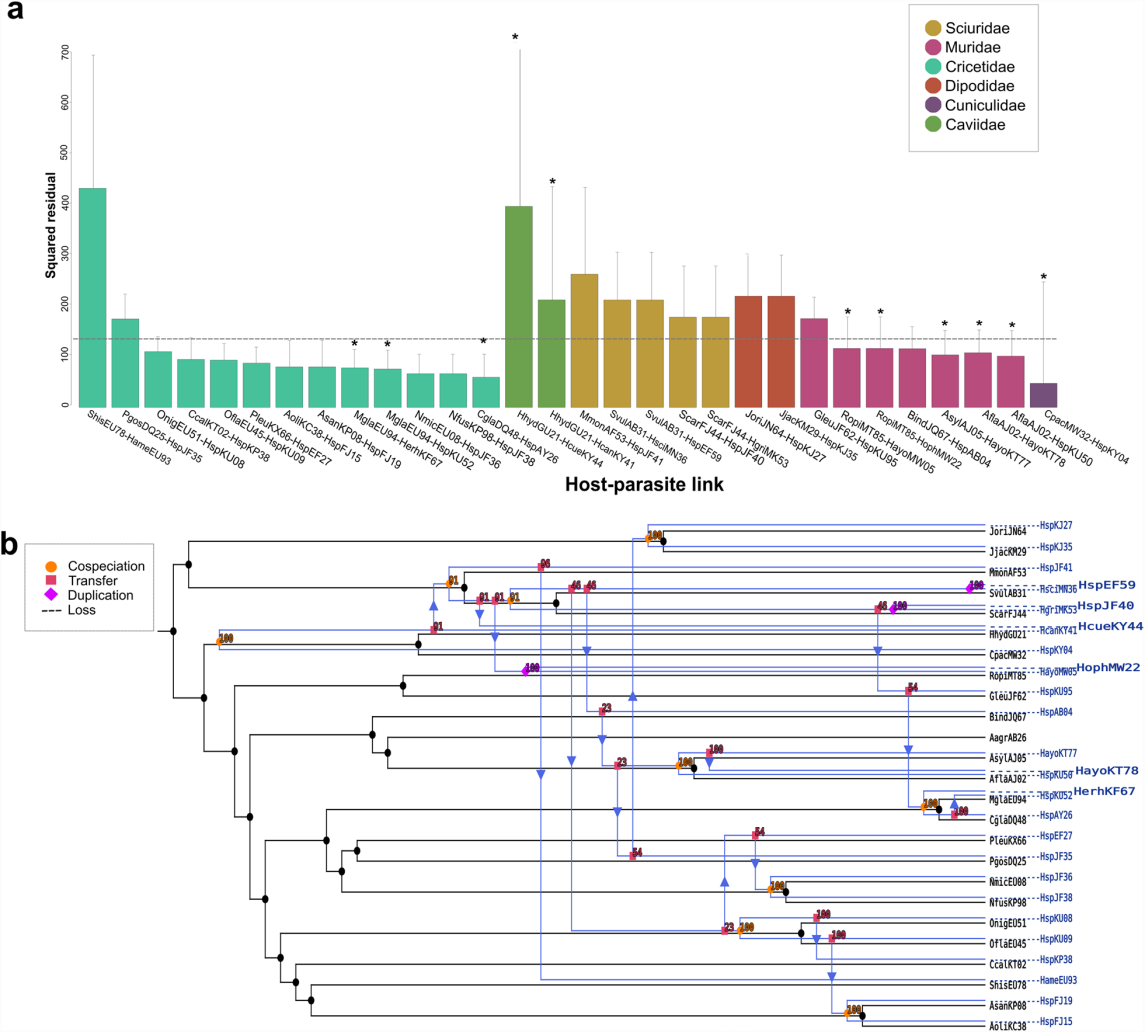
Global-fit and event-based cophylogenetic analysis between *Hepatozoon* and its rodent hosts. **a** Contribution of each *Hepatozoon*–vertebrate host link to the global phylogenetic congruence. Each bar represents the squared residual of each association, and are color-coded according to the host family. Error bars correspond to 95% confidence intervals of the squared residuals. The median squared residual is indicated as a dotted line. Asterisks at the top of each bar represent a significant ParaFitLink1 value. **b** Coevolutionary reconstruction of the host (black lines) and parasite (blue lines) phylogenies with the lowest global cost according to eMPRess

PACo analysis of the *Hepatozoon* spp. sequences obtained from rodent hosts revealed a significant cophylogenetic relationship (*m*^2^_XY_ = 0.530, *P* < 0.001, *R*^2^ = 0.47). The 95% confidence of squared residuals of four host–parasite associations corresponding to the family Cricetidae was below the median square residual value (Fig. 5a). In total, 66% (*n* = 20) of the links registered for rodent hosts were with uncharacterized *Hepatozoon* sequences, followed by *Hepatozoon ayorgbor* (10%, *n* = 3), *H. sciuri*, *H. ophisauri*, *H. griseisciuru*, *H. americanum*, *H. canis*, and *H. erhardovae* (all 3.33%, *n* = 1). The highest squared residual occurred in the association between the hispid cotton rat *Sigmodon hispidus* (ShisEU78) and *H. americanum* (HameEU93), and the lowest was the lowland paca *Cuniculus paca* (CpacMW32) associated with an uncharacterized *Hepatozoon* sp. (HspKY04).

ParaFit also revealed a significant global cophylogenetic relationship with 11 significant links (ParaFitGlobal Statistic = 9.635, *P* = 0.004; Fig. 5a). The highest ParaFitLink1 was for the association between the lowland paca *Cuniculus paca* (CpacMW32) and *Hepatozoon* sp. (HspKY04) (F1 = 2.9502, *P* = 0.006), and the lowest was between the wood mouse *Apodemus sylvaticus* (AsylAJ05) and *H. ayorgbor* (HayoKT77) (F1 = 0.2880, *P* = 0.031). The individual contribution to the ParaFitGlobal Statistic through the ParaFitLink1 values of the order Rodentia indicated that the congruence between phylogenies was mostly accounted for by the family Muridae, with 5 significant links out of 11 (Fig. 5a). Principal coordinate (PCo) plots for the parasite associated with hosts of the order Rodentia suggest a single well-differentiated cluster corresponding to the families Muridae and Cricetidae (Additional file 5B).

The eMPRess analysis pointed to host switching (*n* = 15) as the most frequent event occurring between *Hepatozoon* spp. and Rodentia, followed by cospeciation (*n* = 11) and duplication (*n* = 3) (Fig. 5b). Host switching was observed between an uncharacterized *Hepatozoon* sp. (HspKU08) from the black-footed colilargo *Oligoryzomys nigripes* to the large vesper mouse *Calomys callosus*. Furthermore, cospeciation was predicted in an uncharacterized *Hepatozoon* sp. sequence (HspJF36/HspJF38) found in the woodrat species *Neotoma micropus* and *Neotoma fuscipes,*, and between *H. ayorgbor* (HayoKT77) and *Hepatozoon* sp. (HspKU50) from the wood mice *Apodemus sylvaticus* and *Apodemus flavicollis*.

### *Hepatozoon*–Squamata associations

**Fig. 6:**
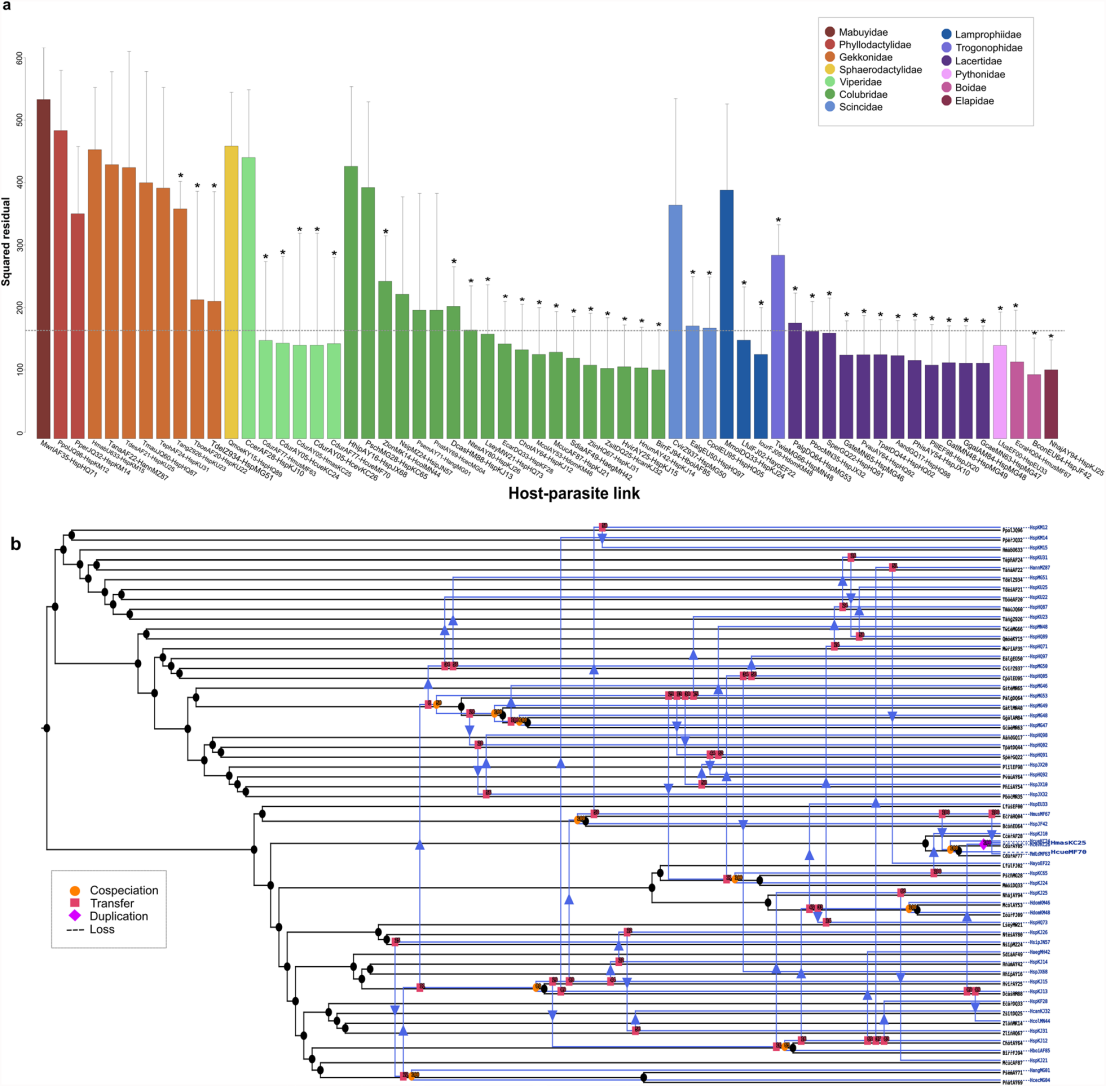
Global-fit and event-based cophylogenetic analysis between *Hepatozoon* and its squamate hosts. **a** Contribution of each *Hepatozoon*–vertebrate host link to the global phylogenetic congruence. Each bar represents the squared residual of each association, and are color-coded according to the host family. Error bars correspond to 95% confidence intervals of the squared residuals. The median squared residual is indicated as a dotted line. Asterisks at the top of each bar represent a significant ParaFitLink1 value. **b** Coevolutionary reconstruction of the host (black lines) and parasite (blue lines) phylogenies with the lowest global cost according to eMPRess

PACo revealed a significant cophylogenetic relationship between parasites and hosts (*m*^2^_XY_ = 0.655, *P* < 0.001, *R*^2^ = 0.35). However, only the 95% confidence of squared residuals of two host–parasite associations were below the median square residual value, which had a significant ParaFitLink1 value (Fig. 6a). These two associations corresponded to an uncharacterized *Hepatozoon* sp. (HspJF42) and *Boa constrictor* (BconEU64), and an uncharacterized *Hepatozoon* sp. (HspKJ25) and *Naja haje* (NhajAY94). Most of the associations within Squamata concerned the families Colubridae (31.7%, *n* = 19), Lacertidae (20%, *n* = 12), and Gekkonidae (13.3%, *n* = 8). Moreover, most Squamata-derived sequences were distributed across two clusters in the phylogenetic tree of *Hepatozoon* spp., which were separated by a group of *Hepatozoon* spp. associated to Rodentia (Fig. 3). One of these clusters included some other sequences derived from different orders, such as Anura, Chiroptera, Peramelemorphia, and Gymnophiona. The highest squared residual was noted for the link between the Wright’s skink *Trachylepis wrightii* (syn. *Mabuya wrightii*) (MwriAF35) and an uncharacterized *Hepatozoon* sp. (HspHQ71), and the lowest squared residuals corresponded to an association between *Boa constrictor imperator* (BconEU64) and *Hepatozoon* sp. (HspJF42). Principal coordinate plots for the parasites associated with hosts of the order Squamata suggested three clusters belonging to the families Lacertidae, Colubridae, and Gekkonidae (Additional file 6B).

A significant cophylogenetic relationship was also revealed by ParaFit (ParaFitGlobal Statistic = 55.033, *P* = 0.001) and 44 host–parasite associations were found to contribute significantly to the ParaFitGlobal Statistic (Fig. 6a). The highest ParaFitLink1 was estimated for the link between Gallot’s lizard *Gallotia galloti* (GgalAM84) and an uncharacterized *Hepatozoon* sp. (HspMG48) (F1 = 4.3568, *P* = 0.001), and between Boettger’s lizard *Gallotia caesaris* (GcaeMN63) and an uncharacterized *Hepatozoon* sp. (HspMG47) (F1 = 4.3568, *P* = 0.001). The lowest ParaFitLink1 value concerned the Caspian whipsnake *Dolichophis caspius* (DcasHM88) and *Hepatozoon* sp. (HspKJ13) (F1 = 1.4314, *P* = 0.005).

The eMPRess analysis positioned most coevolutionary events at higher taxonomic levels. Accordingly, host switching (*n* = 48) was the most frequent event predicted between the *Hepatozoon* spp. and their Squamata hosts, followed by cospeciation (*n* = 10) and duplication (*n* = 1) (Fig. 6b; Table 1). Host switching was calculated from an uncharacterized *Hepatozoon* sp. (HspJX32) from the wall lizard *Podarcis bocagei* to the dwarf lizard *Atlantolacerta andreanskyi*, or from *H. musa* (HmusMF67) of the rainbow boa *Epicrates crassus* to the rattlesnake *Crotalus durissus*. Moreover, cospeciation was predicted in *H. boiga* (HboiAF85) and an uncharacterized *Hepatozoon* sp. sequence (HspKJ12) with *B. irregularis* and the herald snake *Crotaphopeltis hotamboeia*, respectively.

### *Hepatozoon*–invertebrate associations

**Fig. 7:**
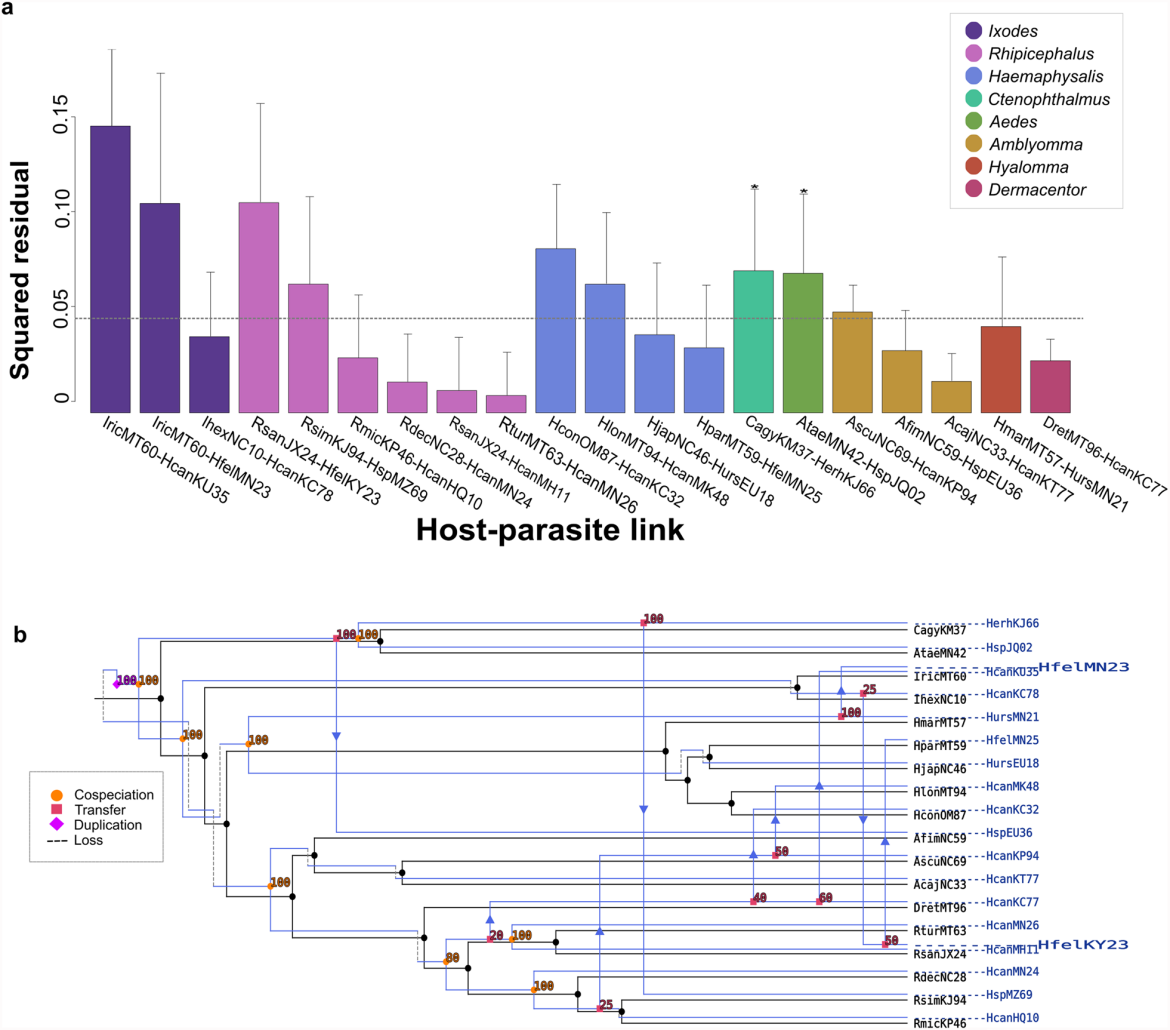
Global-fit and event-based cophylogenetic analysis between *Hepatozoon* and its invertebrate hosts. **a** Contribution of each *Hepatozoon*–invertebrate host link to the global phylogenetic congruence. Each bar represents the squared residual of each association, and are color-coded according to the host family. Error bars correspond to 95% confidence intervals of the squared residuals. The median squared residual is indicated as a dotted line. **b** Coevolutionary reconstruction of the host (black lines) and parasite (blue lines) phylogenies with the lowest global cost according to eMPRess

PACo analysis of *Hepatozoon* spp. associated to invertebrate hosts revealed a significant cophylogenetic relationship (*m*^2^_XY_ = 0.632, *P* < 0.001, *R*^2^ = 0.37) with five links displaying a 95% confidence of squared residuals below the median square residual value (Fig. 7a). Most confirmed associations were retrieved from the tick genus *Rhipicephalus* (15%, *n* = 3), followed by the genus *Amblyomma* (5%, *n* = 1), and *Dermacentor* (5%, *n* = 1). The highest squared residual occurred in the association between *Ixodes ricinus* (IricMT60) and *H. canis* (HcanKU35), and the lowest was recorded for the association between *Rhipicephalus turanicus* (RturMT63) and *H. canis* (HcanMN26). A global nonsignificant cophylogenetic relationship was also revealed by the ParaFit analysis (ParaFitGlobal Statistic = 8.810, *P* = 0.124). Only two links were significant, namely, between an uncharacterized *Hepatozoon* sp. sequence (HspJQ02) and the mosquito *Aedes taeniorhynchus* (AtaeMN42) (F1 = 2.99175, *P* = 0.014), and between *H. erhrardovae* (HerhKJ66) and the flea *Ctenophthalmus agyrtes* (CagyKM37) (F1 = 2.96025, *P* = 0.016).

The individual contribution to the ParaFitGlobal statistic through the squared residual of the parasite–host links indicated that the congruence between phylogenies was given by several tick genera, e.g., *Rhipicephalus*, *Amblyomma*, and *Dermacentor*. But none of the links of these ticks with *Hepatozoon* spp. were supported with a significant ParaFitLink1 result (Fig. 7a). Finally, principal coordinate (PCo) plots for the parasites associated with invertebrate hosts did not show specific clusters (Additional file 7b).

The eMPRess analysis determined that host switching (*n* = 13) was the most frequent event occurring between *Hepatozoon* spp. and invertebrate hosts, followed by cospeciation (*n* = 6) (Fig. 7b). For example, host switching was predicted in *H. erhardovae* (HerhKJ66) from the flea *Ctenophthalmus agyrtes* to the tick *Rhipicephalus simus*.

## Discussion

More than 300 *Hepatozoon* spp. have been described with the use of molecular and morphological methods in a wide range of wild and domesticated animals [30]. Importantly, these apicomplexan parasites are usually nonpathogenic or cause disease with only mild clinical signs [11]. This may be suggestive of a long coevolutionary relationship with their hosts, given the relatively low pathogenicity of these parasites or their adoption of transmission strategies that do not favor high virulence. In this study, the hypothesis of coevolution was tested by using patristic distances of phylogenies obtained from *Hepatozoon* spp. associated with vertebrate and invertebrate hosts of several orders.

The review of *Hepatozoon* DNA sequences deposited in GenBank showed that the most used molecular marker is 18S rDNA. This finding has practical implications owing to the large number of sequences available for phylogenetic and diagnostic purposes and the sufficient differentiation at genus and species level provided by this marker [30]. However, 18S rDNA is highly conserved [46–48], which partially explains why 40.5% of the sequences recorded in GenBank have been identified only to the genus level. Conversely, the complexity and expertise required to perform morphological assessment of parasites in blood and tissue samples contribute to the incomplete characterization of *Hepatozoon* species. Traditional identification techniques such as morphological descriptions of intracellular gamonts often result in misidentification [30], and often fail to differentiate between morphometrically similar species such as *H. clamatae* and *H. catesbianae* [49]. Therefore, the integration of both morphological and molecular methods should improve species identification [50]. The use of mitochondrial genes such as *COI* and *COIII*, and apicoplast markers such as 28S rRNA with higher interspecies resolution, may provide better alternatives for parasite identification, as in some cases they have been successfully used for identification down to the species level [48, 49]. Therefore, the use of additional markers and more comprehensive phylogenetic analyses would improve the discrimination between *Hepatozoon* spp. and allow the identification of haplotypes circulating in different vertebrate and invertebrate hosts [46, 48]. Consequently, this would enable the identification of specific parasite haplotypes affecting different host species in a geographical area, and how these in turn participate in the transmission of the parasites to new susceptible hosts, as previously demonstrated for *H. canis* [51]. Since cophylogenetic analyses were conducted with 18S rDNA parasite phylogenies and with *cytB* mitochondrial host phylogenies, it may possible that the phylogenetic inference obtained herein may vary.

The majority of *Hepatozoon* sequences available have been characterized as *H. canis* and *H. felis*. Moreover, these sequences have derived from studies conducted in both America and Europe, which is consistent with the findings of Thomas et al. [52], in which isolates from hosts of Carnivora and Rodentia were the most common, including *H. canis* and *H. felis* [52]. In addition, the largest number of *Hepatozoon* sequences originated from Brazil and the USA. This may be explained by the differential distribution of *Hepatozoon* spp. in these geographical areas or a bias in the true world distribution of *Hepatozoon* spp. according to research conducted in these regions. Consequently, we anticipate the existence of a diverse array of unidentified *Hepatozoon* species of parasite circulating in wild animals in other geographical areas.

Our data showed that most *Hepatozoon* sequences have been derived from Carnivora and Squamata. This may be explained by the transmission routes of this parasite, which include the ingestion of infected invertebrate hosts, and its trophic transmission, when predators feed on infected prey [53]. In the latter case, prey such as frogs, lizards, and grey squirrels [54] may function as paratenic or first intermediate hosts that are infected with parasitic tissue cysts. For instance, it has been shown that saurophagous snakes, lizards, and frogs can be infected with the same *Hepatozoon* spp., as the predator–prey relationship between these hosts facilitates cross-species transmission [55, 56].

Most GenBank sequences identified to species level corresponded to *H. canis* likely due its widespread distribution in domestic animals [51]. The geographical dispersion of this species may be related to the geographical movement of dogs associated with human migration, since little genetic differentiation of haplotypes has been observed between *H. canis* populations around the world [51]. According to the event-based analyses performed in this study, *H. canis* seems to be a parasite that has undergone frequent host switches among hosts within the family Canidae. Moreover, wild canids may be reservoirs of this parasite species due to its relatively benign course of infection [57].

The order Carnivora seem to have sustained a coevolutionary relationship with their associated *Hepatozoon* spp., mainly with *H. felis* and *H. canis*. Most significant links or associations were obtained with *H. felis* and their hosts within the family Felidae, and between *H. canis* and hosts within the family Canidae, which may explain the subclinical infection associated with feline and canine hepatozoonosis [11, 58]. Importantly, the links between *C. lupus familiaris* and *C. latrans* with *H. americanum*, one of the most pathogenic *Hepatozoon* spp., were not found to be significant. They showed one of the highest squared residuals in the event-based analysis, suggesting a recent switch to these hosts. Therefore, *H. americanum* may be poorly adapted to domestic dogs and coyotes probably due to their recent association. Even though it has been shown that coyotes naturally infected with *H. americanum* may not present a severe clinical picture with characteristic bone lesions [23], the global-fit analyses showed a nonsignificant association [59].

A *Hepatozoon* sp. associated with the raccoon *P. lotor* showed a significant coevolutionary relationship with this host. We note that this sequence was identical to *H. procyonis*, a species occurring in different procyonids from the Americas [60, 61]. In contrast, neither PACo nor ParaFit supported a coevolutionary association between *H. procyonis* and the coati *Nasua nasua*. Nevertheless, eMPRess predicted cospeciation events between this *Hepatozoon* sp. (i.e., most likely *H. procyonis*) and *P. lotor*, and between *H. procyonis* and *N. nasua*. The close phylogenetic relationship between the two hosts [62] may account for this pattern.

The phylogenetic tree of *Hepatozoon* sequences derived from hosts of the order Squamata consisted of two polyphyletic groups separated by a monophyletic cluster formed by *Hepatozoon* from rodents. In addition, sequences obtained from other host orders were intermixed with one of the two clusters, thus suggesting that rodents, bats, or frogs may serve as first intermediate hosts of *Hepatozoon*, which has been explored experimentally with *H. ayorgbor* [63]. In addition, all cospeciation events predicted between *Hepatozoon* obtained from squamates occurred among closely related host species. This indicates that coevolution between these apicomplexan parasites with snakes, geckos, or other lizards is probably recent. However, coevolution is favored by long-term interactions and adaptations, as in the case of the vector-borne bacteria *Bartonella* when compared with non-vector transmitted *Leptospira* spp. [64]. Therefore, a time-calibrated phylogenetic tree would be needed to test this hypothesis rigorously.

Host switches need to be interpreted with caution, since the detection of *Hepatozoon* DNA in a host’s blood does not necessarily imply that the animal is an intermediate host and part of the parasite’s life cycle. For instance, a host switch of *Hepatozoon* sp. was predicted from *P. lotor* to the pale fox *V. pallida*. This link between *Hepatozoon* sp. with *V. pallida* was not significant according to PACo and the ParaFit function, however PACo does not strictly test the significance of host–parasite links. Therefore, the interpretation of coevolutionary events should consider both cophylogenetic results and event-based predictions. This apparent host switch may be explained by a pale fox [65] preying on an invertebrate host infected with a *Hepatozoon* sp. originally associated with a raccoon.

Phylogenies between *Hepatozoon* spp. and hosts of the orders Carnivora, Rodentia, and Squamata were found to be globally congruent, which may explain why *Hepatozoon* spp. cause subclinical or mild infections in their hosts from these orders [2, 66]. Clinical signs associated with *Hepatozoon* infections are well documented in domesticated animals, but in wildlife, this protozoa is usually found without associated disease, such as *Hepatozoon apri* in the Japanese wild boar *Sus scrofa leucomystax* [67], an uncharacterized *Hepatozoon* sp. in the giant panda *Ailuropoda melanoleuca* [68], or causing mild blood cell alterations, such as dehemoglobinization or karyolysis produced by *Hepatozoon angeladaviesae* in *Philothamnus* snakes [69]. The mild clinical presentation associated with hepatozoonosis may be due to the evolutionary dependence of the parasite on its host, since a balance is established between the parasite’s attack and the host’s immune defense [70, 71]. *Hepatozoon* spp. can therefore be well adapted to their hosts, i.e., the little damage inflicted to its hosts could hypothetically translate into an advantage for the parasite for effective transmission to the next one [72].

Global phylogenetic congruence was obtained between *Hepatozoon* sequences associated to invertebrate hosts by PACo, but not with the ParaFit function. While the reasons for this discrepancy are unclear, in some situations PACo tends to be less conservative than ParaFit [26]. PACo is more prone to making type I errors or is more likely to refuse a true null hypothesis, which would translate as a false congruence between phylogenies. However, the R^2^ values supports these findings since it suggests that there is congruence between the phylogenies. In addition, a nonsignificant association should be expected because the invertebrate host acts as a vector. In this case, host switching could be advantageous for enhancing parasite transmission, particularly when there is no strong physiological dependence of the parasite upon the intermediate host. However, it should also be noted that the low number of *Hepatozoon* sequences derived from invertebrate hosts in the study makes it difficult to draw reliable conclusions.

*Hepatozoon* spp. have the potential to cause emerging diseases in wildlife animals [73], which conforms with the frequent host switch events observed in this study. Generally, when incongruence is observed between parasite and host phylogenies, it is expected to result from evolutionary events such as host switching, duplication, or loss [74]. On the contrary, a high level of cospeciation contributes to congruence between phylogenies of hosts and parasites [75]. Host switching in *Hepatozoon* may be favored by high parasite dispersal due to the wide distribution of invertebrate and vertebrate hosts [76–78]. Even more so, the invertebrate hosts associated with *Hepatozoon* spp. usually exhibit a generalist feeding behavior [27, 79], which would facilitate host switching [77]. In addition, *Hepatozoon* spp. tend to infect vertebrate hosts of the same taxonomic family, as evidenced in our results. However, some authors argue that the vertebrate host ecology is more important than their phylogeny in determining whether a host is susceptible to infection by a particular species [8, 56]. If prey and predators are exposed to the same habitat they will be exposed to the same infected vectors and could become infected by the same species [65].

## Conclusions

The present study demonstrated that the phylogeny of *Hepatozoon* spp. is overall congruent with the phylogeny of its vertebrate hosts. However, host switching has been a recurring event in shaping the evolutionary history of this host–parasite system. This finding is important to better understand the emergence by spillover of hepatozoonosis in new susceptible host species in specific geographical areas [80, 81]. Furthermore, this insight facilitates the development of conservation strategies by identifying potential reservoirs of the disease [82]. However, the present study did not evaluate other factors limiting host switching, such as geographical barriers or the proximity required between the new host and the original one to enable host switching. Thus, future research should study the phylogeography of other *Hepatozoon* spp. In particular, further work is required to identify whether predicted haplotypes are differentially distributed according to their geographical origin and which of them are prone to infect hosts related to those already described. Furthermore, the present study used two distinct genes to compare phylogenies of parasites and their hosts; therefore, phylogenetic inference could vary if different genes were analyzed, as evolutionary histories may differ among loci. Therefore, future studies should assess the effect of the molecular marker on the robustness and accuracy of cophylogenetic studies of *Hepatozoon*.

## Supplementary Information

Additional file 1: Database of *Hepatozoon* spp. associated to vertebrate and invertebrate hosts.

Additional file 2: Event cost landscapeplot and MPR total cost histogram of *Hepatozoon* spp. associated with carnivores, rodents, squamata, and invertebrates.

Additional file 3: Scripts used for running the PACo and Parafit analyses in R

Additional file 4: **a** Bayesian inference phylogenetic tree of carnivore hosts of *Hepatozoon* spp. used in the analysis. Posterior probability values are indicated next to each node. Line width, node size and color are proportional to posterior probabilities. Each host is identified by the corresponding Genbank sequence accession number and code used in the analysis. **b** Procrustean superimposition plot between the principal coordinates derived from patristic distances of the 18S of *Hepatozoon* spp. and their carnivore host phylogenies. Parasiteand hostcodes are denoted as circles and arrow heads, respectively.

Additional file 5: **a** Bayesian inference phylogenetic tree of rodent hosts of *Hepatozoon* spp. used in the analysis. Posterior probability values are indicated next to each node. Line width, node size and color are proportional to posterior probabilities. Each host is identified by the corresponding Genbank sequence accession number and code used in the analysis. **b** Procrustean superimposition plot between the principal coordinates derived from patristic distances of the 18S of *Hepatozoon* spp. and their rodent host phylogenies. Parasiteand hostcodes are denoted as circles and arrow heads, respectively.

Additional file 6: **a** Bayesian inference phylogenetic tree of Squamata hosts of *Hepatozoon* spp. used in the analysis. Posterior probability values are indicated next to each node. Line width, node size and color are proportional to posterior probabilities. Each host is identified by the corresponding Genbank sequence accession number and code used in the analysis. **b** Procrustean superimposition plot between the principal coordinates derived from patristic distances of the 18S of *Hepatozoon* spp. and their squamata host phylogenies. Parasiteand hostcodes are denoted as circles and arrow heads, respectively.

Additional file 7: **a** Bayesian inference phylogenetic tree of invertebrate hosts of *Hepatozoon* spp. used in the analysis. Posterior probability values are indicated next to each node. Line width, node size and color are proportional to posterior probabilities. Each host is identified by the corresponding Genbank sequence accession number and code used in the analysis. **b** Procrustean superimposition plot between the principal coordinates derived from patristic distances of the 18S of *Hepatozoon* spp. and their invertebrate host phylogenies. Each parasite and host are denoted as circles and arrow heads, respectively.

## Data Availability

Data and scripts used for running all analyses are provided in the Additional Files section.
