# Supplementary figures and images for: Host switching is the main driver of coevolution between Hepatozoon parasites and their vertebrate hosts
Source: Parasit Vectors. 2025 Jul 23;18:293. doi: 10.1186/s13071-025-06870-4 (PMC12288290; doi:10.1186/s13071-025-06870-4)

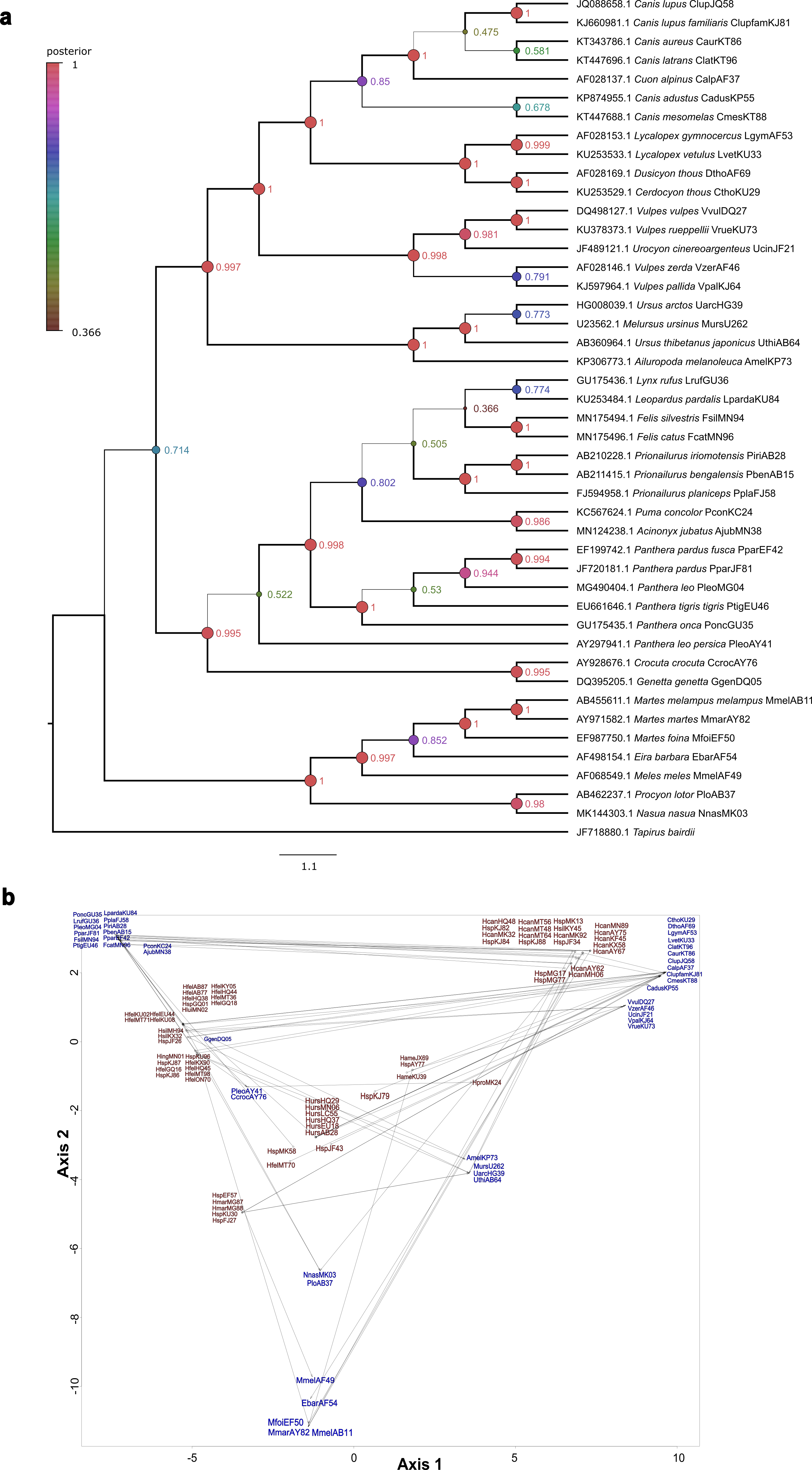

Supplement: Supplementary file 4 — Additional file 4: a Bayesian inference phylogenetic tree of carnivore hosts of Hepatozoon spp. used in the analysis. Posterior probability values are indicated next to each node. Line width, node size and color are proportional to posterior probabilities. Each host is identified by the corresponding Genbank sequence accession number and code used in the analysis. b Procrustean superimposition plot between the principal coordinates derived from patristic distances of the 18S of Hepatozoon spp. and their carnivore host phylogenies. Parasiteand hostcodes are denoted as circles and arrow heads, respectively. [file 13071_2025_6870_MOESM4_ESM.png]

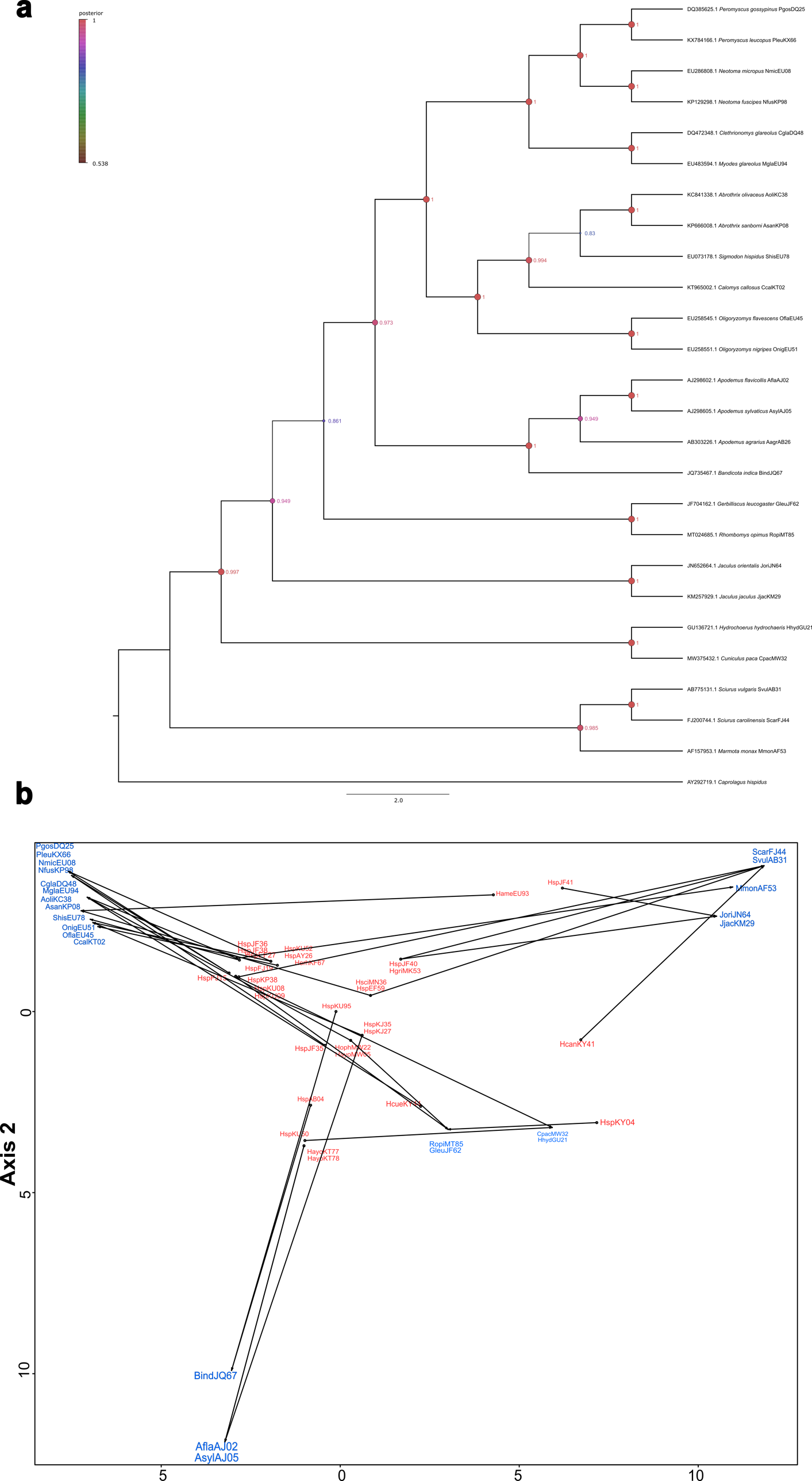

Supplement: Supplementary file 5 — Additional file 5: a Bayesian inference phylogenetic tree of rodent hosts of Hepatozoon spp. used in the analysis. Posterior probability values are indicated next to each node. Line width, node size and color are proportional to posterior probabilities. Each host is identified by the corresponding Genbank sequence accession number and code used in the analysis. b Procrustean superimposition plot between the principal coordinates derived from patristic distances of the 18S of Hepatozoon spp. and their rodent host phylogenies. Parasiteand hostcodes are denoted as circles and arrow heads, respectively. [file 13071_2025_6870_MOESM5_ESM.png]

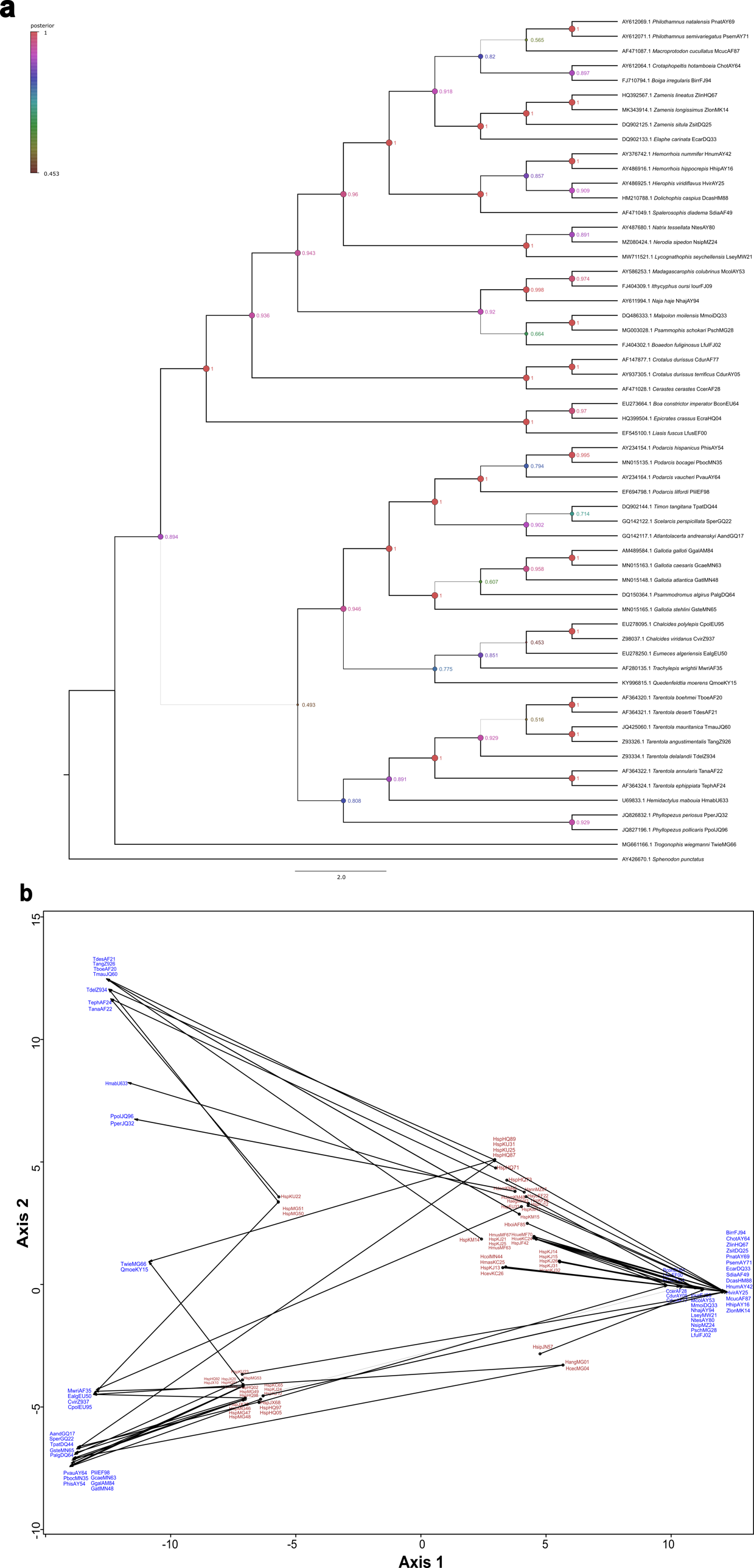

Supplement: Supplementary file 6 — Additional file 6: a Bayesian inference phylogenetic tree of Squamata hosts of Hepatozoon spp. used in the analysis. Posterior probability values are indicated next to each node. Line width, node size and color are proportional to posterior probabilities. Each host is identified by the corresponding Genbank sequence accession number and code used in the analysis. b Procrustean superimposition plot between the principal coordinates derived from patristic distances of the 18S of Hepatozoon spp. and their squamata host phylogenies. Parasiteand hostcodes are denoted as circles and arrow heads, respectively. [file 13071_2025_6870_MOESM6_ESM.png]

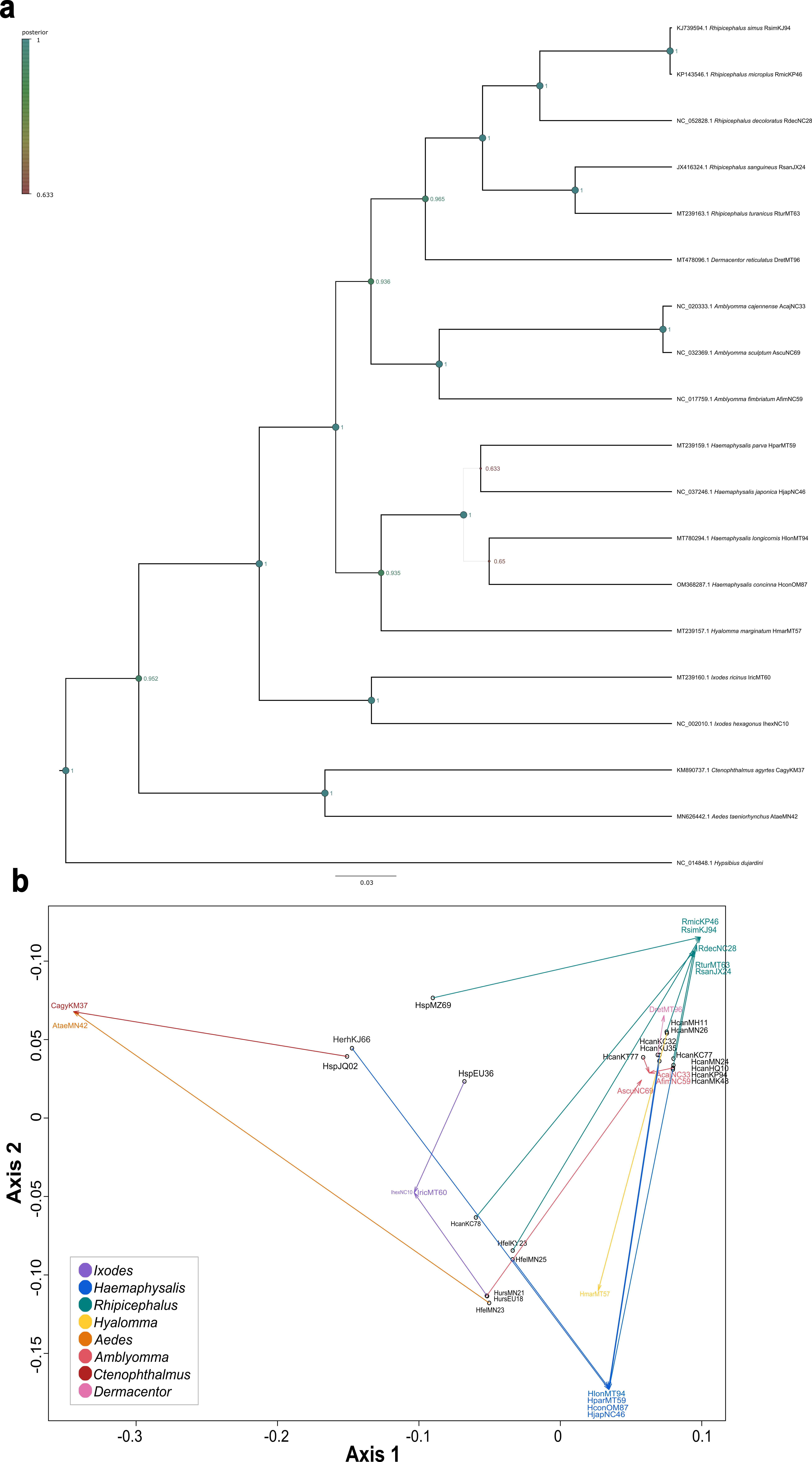

Supplement: Supplementary file 7 — Additional file 7: a Bayesian inference phylogenetic tree of invertebrate hosts of Hepatozoon spp. used in the analysis. Posterior probability values are indicated next to each node. Line width, node size and color are proportional to posterior probabilities. Each host is identified by the corresponding Genbank sequence accession number and code used in the analysis. b Procrustean superimposition plot between the principal coordinates derived from patristic distances of the 18S of Hepatozoon spp. and their invertebrate host phylogenies. Each parasite and host are denoted as circles and arrow heads, respectively. [file 13071_2025_6870_MOESM7_ESM.png]
